# Achieving a Good Life Time in a Vertical-Organic-Diode Gas Sensor

**DOI:** 10.3390/s140916287

**Published:** 2014-09-02

**Authors:** Ming-Zhi Dai, Yen-Ho Chen, Ming-Yen Chuang, Hsiao-Wen Zan, Hsin-Fei Meng

**Affiliations:** 1 Institute of Physics, National Chiao Tung University, Hsinchu 300, Taiwan; E-Mails: whiffet_tw@hotmail.com (M.-Z.D.); patrickyoyo0624@hotmail.com (Y.-H.C.); meng@mail.nctu.edu.tw (H.-F.M.); 2 Department of Photonics and Institute of Electro-Optical Engineering, National Chiao Tung University, Hsinchu 300, Taiwan; E-Mail: a524524524@livemail.tw

**Keywords:** ammonia, gas sensor, polythiophene, solid-state sensors

## Abstract

In this study, we investigate the keys to obtain a sensitive ammonia sensor with high air stability by using a low-cost polythiophene diode with a vertical channel and a porous top electrode. Poly(3-hexylthiophene) (P3HT) and air-stable poly(5,5′-bis(3-dodecyl-2-thienyl)-2,2′-bithiophene) (PQT-12) are both evaluated as the active sensing layer. Two-dimensional current simulation reveals that the proposed device exhibits numerous connected vertical nanometer junctions (VNJ). Due to the de-doping reaction between ammonia molecules and the bulk current flowing through the vertical channel, both PQT-12 and P3HT VNJ-diodes exhibit detection limits of 50-ppb ammonia. The P3HT VNJ-diode, however, becomes unstable after being stored in air for two days. On the contrary, the PQT-12 VNJ-diode keeps an almost unchanged response to 50-ppb ammonia after being stored in air for 25 days. The improved storage lifetime of an organic-semiconductor-based gas sensor in air is successfully demonstrated.

## 1. Introduction

Solid-state gas sensors have drawn considerable attention for their applications in environmental pollution monitoring [1], toxic or explosive gas detection [2], food condition tracking [3] and non-invasive diagnostics through breath analysis [4–6]. Among these applications, sensors based on organic semiconductor (OSC) materials are particularly promising because of their low-cost process, room-temperature operation and wide selection of material properties [4,7]. However, when OSC materials are stored in air, oxidation due to oxygen and moisture is known to destroy the electric property in OSC materials within a few days [8,9]. Recently, researchers proposed various air-stable OSC materials and successfully demonstrated air-stable organic thin-film transistors (OTFTs) [10–12]. A 120-day storage time was reported for OTFT with an air-stable OSC layer. In an OSC-based gas sensor, however, the air stability after long-term storage has been not reported yet.

In this work, we studied the air stability of a sensitive ammonia gas sensor based on a vertical organic diode. Two kinds of polythiophene materials are used in the proposed sensor. One is poly(3-hexylthiophene) (P3HT); the other is air-stable poly(5,5′-bis(3-dodecyl-2-thienyl)- 2,2′-bithiophene) (PQT-12). In recent years, abundant research focused on developing nanostructure gas sensors [13–16]. Sensors with a nanostructure can increase the surface-to-volume ratio and, thus, lead to higher performance. Chen shows that under ultraviolet light illumination during gas sensing, single-walled carbon nanotubes can detect ammonia, nitric oxide and nitrogen dioxide in parts per trillion level [17]. Besides, Hassan reports that vertical zinc oxide nanorod arrays exhibit high sensitivity to hydrogen [18]. Here, instead of changing the sensing layer into a nanostructure, we develop an electrode with nanopores to form vertical nano-channels in the sensing layer. In our previous work, we had used a P3HT-based vertical diode with a porous top electrode to detect the breath ammonia of rats [19]. The vertical diode exhibits numerous vertical nanometer junctions and is named the vertical nanometer junction (VNJ)-diode. In this work, we demonstrate that the P3HT VNJ-diode exhibits air stability only for 1–2 days. Using PQT-12 to replace P3HT, we successfully realize a sensitive ammonia sensor with a detection limit of 50 ppb after being stored in air for 25 days. In a previous report, a resistor-type sensor using PQT-12 film was found to have almost no response to 1000 ppm ammonia [20]. In that work, the addition of carbon nanotubes (CNTs) into PQT-12 can greatly improve the sensing response. In this work, we demonstrate that using the VNJ-diode structure, the bulk current flowing through PQT-12 responds to 50-ppb ammonia. Together with the simple structure and low-cost process, the improved air stability demonstrated in this work facilitates the future commercialization of OSC-based gas sensors.

## 2. Experimental Section

**Figure 1.**
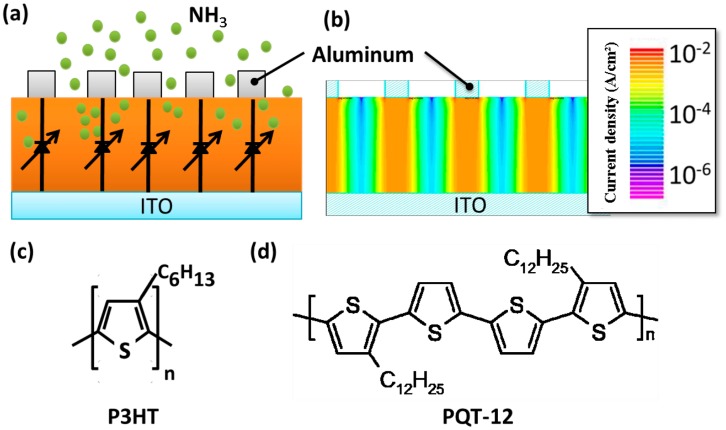
(**a**) The structure of the polythiophene vertical nanometer junction (VNJ)-diode; (**b**) the simulated current distribution of the poly(3-hexylthiophene) (P3HT) VNJ-diode. The molecular structures of (**c**) P3HT and (**d**) poly[5,5′-bis(3-dodecyl-2-thienyl)- 2,2′-bithiophene] (PQT-12).

The structure of the proposed VNJ-diode is shown in Figure 1a. Figure 1b shows the simulated current distribution of the P3HT VNJ-diode. A Silvaco TCAD simulator (Silvaco Inc., Santa Clara, CA, USA) was used to perform the simulation with the parameters provided in [21]. The molecular structures of two polythiophene-based organic semiconductor materials, P3HT and PQT-12, are shown in Figure 1c,d, respectively.

**Figure 2.**
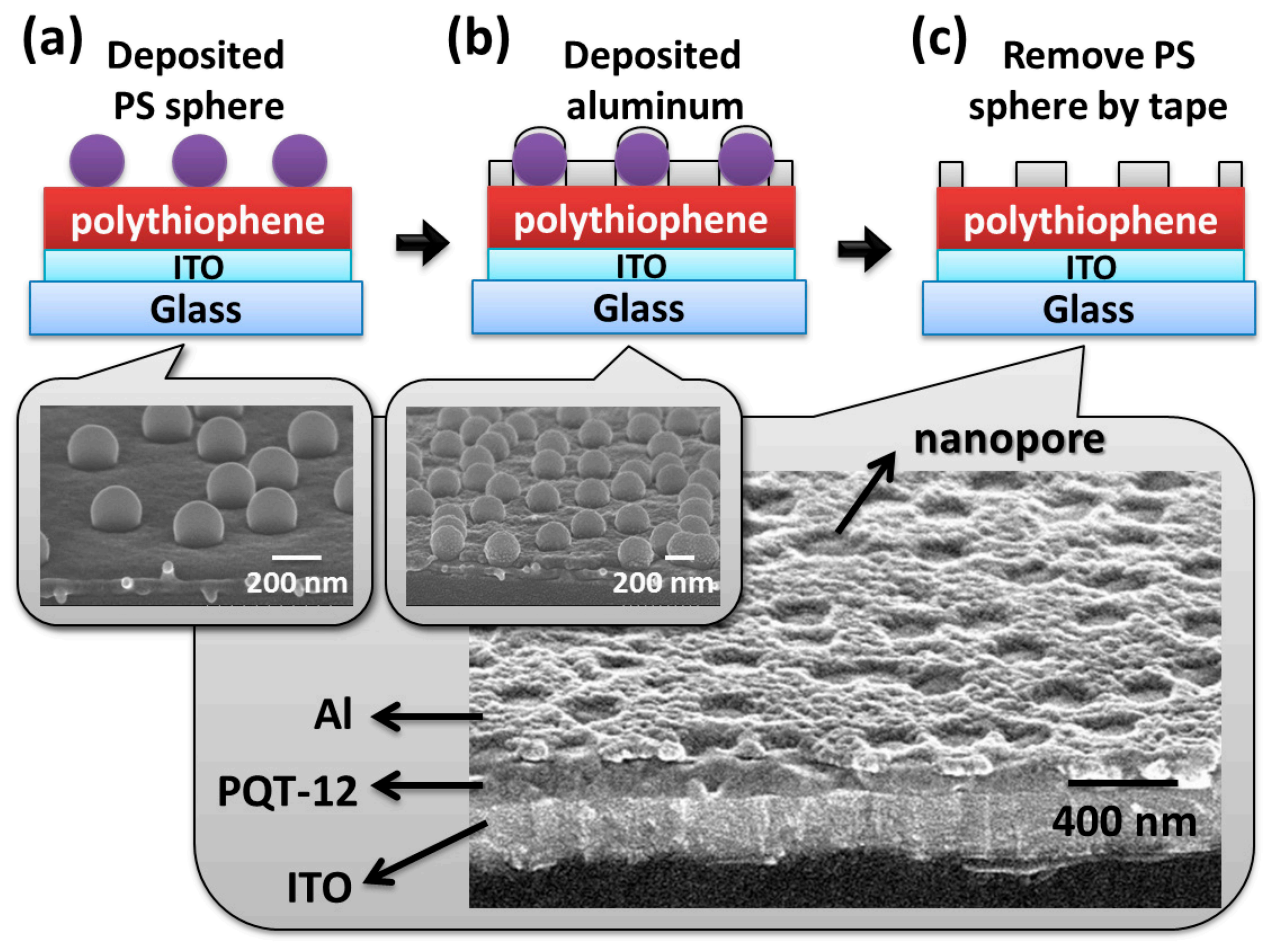
The fabrication process of the P3HT VNJ-diode. (**a**) Polystyrene (PS) spheres adsorbed onto P3HT; (**b**) aluminum of 40 nm was deposited as the metal electrode; and (**c**) the schematic diagram and the SEM image of the top electrode with nano-pores.

Figure 2 shows the fabrication process of the VNJ-diode. A glass substrate with patterned bottom electrode, indium tin oxide (ITO), is prepared (purchased from Dong Guang, resistivity 7 Ω/square). The ITO electrode is treated by 100 W oxygen plasma for 15 min. For the PQT-12 VNJ-diode, PQT-12 (American Dye Source, Inc., Quebec, Canada, molecular weight 15,000–50,000) material dissolved in chloroform (purchased from Aldrich, St. Louis, MO, USA) (0.8 wt%) was spun coated on a substrate to form a PQT-12 layer. After the PQT-12 film was annealed at 140 °C for 30 min, the thin-film PQT-12 was spin-rinsed with *p*-xylene (purchased from Aldrich). The resulting PQT-12 layer exhibits a thickness of 60–100 nm. The substrate was then submerged into a dilute ethanol solution of negatively charged polystyrene (PS) spheres (Fluka). PS spheres with a diameter of 200 nm were adsorbed on the substrate as the shadow mask. Optimized sphere densities (about 5 #/μm^2^) were obtained by using the concentration of the PS sphere as 0.24 wt% with 40 s soaking time. The wet substrate was dipped into boiling isopropyl alcohol (IPA) for 10 s. The substrate was blow-dried immediately after dipping into IPA. Aluminum (Al) of 40 nm was thermal evaporated as the top electrode with an active area of 1 mm^2^. Adhesive tape (Scotch, 3 M) was used to remove the PS spheres. An aluminum top electrode with high-density nanopores was formed. For the P3HT VNJ-diode, P3HT (Rieke Metals, 2.5 wt% in chlorobenzene, molecular weight 50,000–70,000) was spun coated on ITO substrate. The P3HT film was annealed at 200 °C for 10 min. After the P3HT layer was spin-rinsed with *p*-xylene, a film thickness of 40 nm was formed. The optimized PS sphere density (about 5 #/μm^2^) can be also obtained by using ethanol with the 0.24 wt % PS sphere and 40-s soaking time.

The PQT-12 VNJ-diode or the P3HT VNJ-diode was placed in a micro-fluid sensing chamber containing a high purity (99.9999%) nitrogen gas. We used an electrical syringe pump to inject the 100 ppm ammonia (NH3) into a tube to mix with high-purity nitrogen gas. The nitrogen gas flow was controlled by a mass-flow controller, and the ammonia concentration was obtained by adjusting the injection speed of the syringe pump. The gas mixture then entered the micro-fluid system.

## 3. Results and Discussion

The simulated two-dimensional current distribution of the VNJ-diode is shown in Figure 1b [21]. Because of the low conductivity of polythiophene materials, such as P3HT or PQT-12, the vertical current flows are limited within the regions with overlapping top and bottom electrodes. Hence, the VNJ-diode operates like vertical nanometer junctions connected in parallel.

**Figure 3.**
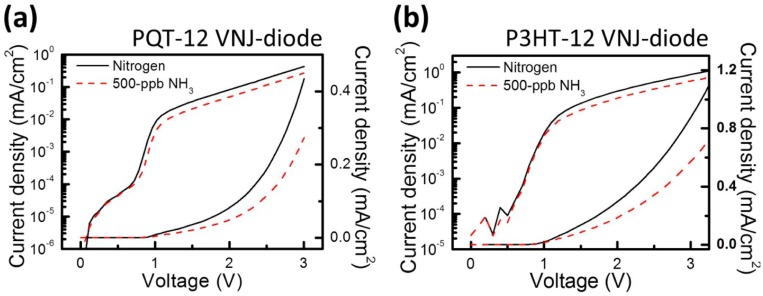
The current densities as a function of the applied bias (J–V) of the (**a**) PQT-12 VNJ-diode and (**b**) the P3HT VNJ-diode before ammonia sensing (solid line) and after 200-s, 500-ppb ammonia sensing (dashed line).

Figure 3a shows the current densities as a function of the applied bias (J–V) of the PQT-12 VNJ-diode before ammonia sensing (solid line) and after 200-s, 500-ppb ammonia sensing (dashed line). Those of the P3HT VNJ-diode are shown in Figure 3b. Because the highest-occupied molecular-orbital (HOMO) level of PQT-12 is 5.3 eV, hole injection from oxygen-plasma-treated ITO (the work function is about 5 eV) to PQT-12 exhibits an energy barrier. Hence, in Figure 3a, the current density at low biasing voltage is small. For the P3HT VNJ-diode, the hole injection barrier is small, since the HOMO level of P3HT (5 eV) is similar to the work function of oxygen-plasma-treated ITO. Regardless of the different hole injection conditions, both the P3HT and PQT-12 VNJ-diodes exhibit obvious current drops after ammonia exposure, because ammonia molecules act as de-doping agents to reduce the carrier concentration in polythiophene-based OSCs [22,23].

**Figure 4.**
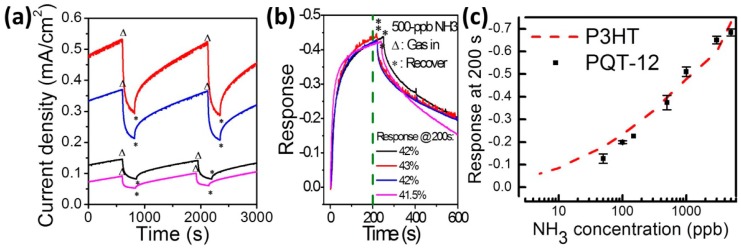
(**a**) The real-time current variations of the fresh PQT-12 VNJ-diode when detecting 500-ppb ammonia. Triangle symbols and star symbols mark the injection and the removal of 500-ppb ammonia, respectively; (**b**) The sensing response of the four devices; and (**c**) the response of the fresh PQT-12 VNJ-diode and the P3HT VNJ-diode as a function of ammonia concentration.

The real-time current variations of fresh PQT-12 VNJ-diode when detecting 500-ppb ammonia are shown in Figure 4a. Four devices fabricated in different runs were measured. When 500-ppb ammonia is injected into the sensing chamber (marked by the triangle symbols), a significant current drop is observed. After removing the ammonia (marked by the star symbols), the current drop can be recovered after about 800–1000 s. The sensing and recovery responses can be repeatedly obtained when 500-ppb ammonia is injected into and removed from the sensing chamber repeatedly. It is noted that devices exhibit device-to-device variation, because of the slightly difference in P3HT film thickness and nanopore density on the top electrode. Such a device-to-device variation, however, can be greatly suppressed when using a current variation ratio to represent the sensing response, as shown in Figure 4b. The current variation ratio, (*I*−*I*_0_)/*I*_0_, is defined as the current minus the initial current (*I*_0_) divided by *I*_0_. The responses of four devices are all about 42% under exposure of ammonia for 200 s. With a long enough recovering time (e.g., 800 s), an almost full recovery can be obtained (as shown in Figure 4a). Finally, with a fixed reading time of 200 s, the sensing response of the fresh PQT-12 VNJ-diode as a function of ammonia concentration is shown in Figure 4c. Data with error bars were obtained from four independent devices. A detection limit of 50 ppb ammonia is obtained in the proposed device. Data obtained from the fresh P3HT VNJ-diode are also represented by the dashed line in Figure 4c. The relationship between the response of VNJ-diode and ammonia concentration is not a linear relationship, but closer to a power-law relationship [24,25].

**Figure 5.**
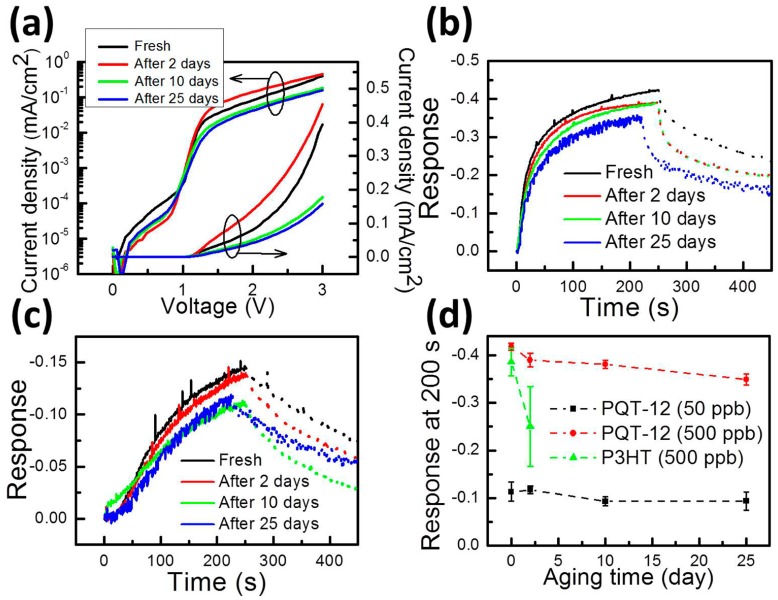
(**a**) The J-V curves of the fresh and aged PQT-12 VNJ-diode. The responses of the fresh and aged sensor as a function of sensing time to (**b**) 500-ppb and (**c**) 50-ppb ammonia. (**d**) The sensor lifetimes of the P3HT VNJ-diode and the PQT-12 VNJ-diode.

So far, we show that the proposed PQT-12 and P3HT VNJ-diodes can detect ammonia molecules in the ppb-regime. The lifetime of P3HT in air, however, is known to be limited. When the P3HT VNJ-diode is exposed to air for two days, the sensor current exhibits a five-times increase at a 2-V bias, and the response to 500-ppb ammonia becomes unstable (shown later in Figure 5d). For real applications, the sensor stability in air needs to be improved. Here, we demonstrate a good air-stability in the OSC-based gas sensor by using PQT-12 to serve as the sensing layer. PQT-12 exhibits the same conjugated backbone as P3HT, but has a better immunity to the humidity-related oxidation in air [26,27]. The J–V curves of the fresh and aged PQT-12 VNJ-diode are shown in Figure 5a. The black J–V curve is measured right after fabricating the device. Red, green and blue J–V curves are measured after putting the PQT-12 VNJ-diode in air for 2, 10 and 25 days, respectively. In Figure 5a, the J–V curves of the PQT-12 VNJ-diode change a bit after being stored in air for several days. However, such a change in the J–V curve does not significantly influence the sensor response to ammonia. The responses of the fresh and aged sensor as a function of sensing time to the 500-ppb and 50-ppb ammonia concentrations are shown in Figure 5b,c, respectively. The dashed line represents the recovery behavior after removing ammonia from the micro-fluid system.

The sensor lifetimes of the P3HT VNJ-diode and the PQT-12 VNJ-diode are compared in Figure 5d. The sensors response readings at 200 s are plotted as a function of aging time. The P3HT VNJ-diode fails to deliver a stable response to 500-ppb ammonia after being stored in air for two days. For the PQT-12 VNJ-diode, quantitatively speaking, the responses to 500-ppb ammonia are −0.41 and −0.35 for the fresh sensor and the 25-day aged sensor. The responses to 50-ppb ammonia are −0.13 and −0.11 for the fresh sensor and 25-day aged sensor. About a 15%–16% degradation is observed after 25 days. However, a clear difference between the responses to the 50-ppb and 500-ppb ammonia concentrations can still be obtained. In particular, for low-concentration applications, such as breath ammonia detection [6], the PQT-12 VNJ-diode is able to detect 50-ppb ammonia with an almost unchanged response after being stored 25 days in air.

## 4. Conclusions

In conclusion, this study presents a sensitive ammonia sensor based on an air-stable organic vertical diode. It is known that many organic semiconductor materials suffer from a short lifetime in air due to the oxidation effect between oxygen, moisture and organic molecules. In this work, we investigated the lifetime of an organic-based ammonia sensor in air. The proposed sensitive ammonia sensor exhibited numerous vertical nanometer junctions (VNJ). Two kinds of polythiophene materials, P3HT and air-stable PQT-12, were used as the organic sensing layer. Using low-cost colloidal lithography (*i.e.*, using self-assembled PS nanospheres as a hard mask), a porous top electrode was fabricated to allow ammonia molecules to diffuse easily into the bulk of the polythiophene sensing layer. The bulk current flowing through vertical nano-junctions (VNJ) was decreased due to the de-doping reaction between ammonia and the polythiophene material. When the P3HT VNJ-diode exhibits a detection limit to 50-ppb ammonia, after two days of being stored in air, the P3HT VNJ-diode becomes unstable due to the oxidation effect. On the other hand, the PQT-12 VNJ-diode exhibits a detection limit of 50-ppb ammonia after being stored in air for 25 days. It emerged that, during the 25-day storage time, the current of the PQT-12 VNJ-diode (biased at 2 V) changes significantly (*i.e.*, from 0.13 to 0.04 mA/cm^2^). The response to 50-ppb ammonia, represented by the current variation ratio, remained almost unchanged during the 25 storage days (*i.e.*, from 0.13 to 0.11). Along with the simple structure and low-cost process, the improved air stability demonstrated in this work facilitates future commercialization of OSC-based gas sensors. In our previous work, we already demonstrated that the proposed polythiophene sensor exhibits a significant response to ammonia and has almost no response to carbon dioxide, acetone and ethanol [19]. In future work, it is expected that the proposed sensor may also be able to detect other kinds of amine-based gas molecules for applications in environmental air pollution detection. Furthermore, by using different kinds of sensing materials in the proposed sensor, we may form a sensing array to further improve the sensing selectivity, as well as the sensing sensitivity.
